# HDAC6-mediated PFKL deacetylation enhances aerobic glycolysis and promotes VSMC proliferation

**DOI:** 10.1016/j.jbc.2025.111075

**Published:** 2025-12-18

**Authors:** Zhao-Kun Hu, Zhi-Yan Ren, Jie-Xin Pang, Hui Li, Meng-Nan Yang, Li-Hua Dong

**Affiliations:** Key Laboratory of Vascular Biology of Hebei Province, Key Laboratory of Neural and Vascular Biology of Ministry of Education, Department of Biochemistry and Molecular Biology, College of Basic Medicine, Hebei Medical University, Shijiazhuang, Hebei, China

**Keywords:** VSMC proliferation, aerobic glycolysis, PFKL, acetylation

## Abstract

Post-translational modifications (PTMs) of the glycolytic enzyme phosphofructokinase, liver type (PFKL) play a vital role in regulating its activity and function. Recently, we observed a reduction of PFKL acetylation in platelet-derived growth factor (PDGF)-BB-induced synthetic vascular smooth muscle cells (VSMCs). However, the function of acetylated PFKL has not been defined. This study aims to elucidate the effects and mechanisms of PFKL acetylation on the development and progression of vascular diseases. We found that the expression of PFKL is upregulated and its acetylation level is decreased in PDGF-BB-induced proliferative VSMCs. HDAC6, which acts as the deacetylase of PFKL, could interact with PFKL to enhance activity of PFKL by accelerating PFKL tetrameric formation and the aerobic glycolysis process, thereby promoting VSMC proliferation, which can be hindered through the application of HDAC inhibitor Trichostatin A (TSA) or siHDAC6. Site prediction and experimental validation revealed that K563 was the main PFKL acetylation site. The recombinant adenoviral vector carrying the PFKL K563R mutant aggravated, while the K563Q mutant attenuated PDGF-BB-induced VSMC proliferation and ligation-induced neointimal formation. Thus, PFKL may be a potential target for vascular reconstruction disease treatment.

Vascular smooth muscle cells (VSMCs) show remarkable plasticity that allows them to dedifferentiate in response to various pathological stimuli such as PDGF-BB, angiotensin II (Ang II) (1).Generally, differentiated VSMCs exhibit a contractile phenotype, whereas dedifferentiated VSMCs exhibit a proliferative or synthetic phenotype characterized by an enhanced rate of proliferation, migration and increased secretion of extracellular matrix (2, 3). VSMC phenotypic switching is a fundamental pathogenesis in various cardiovascular disorders (CVDs) such as atherosclerosis, restenosis, and intimal hyperplasia (4, 5). However, the molecular mechanisms that maintain the VSMC synthetic phenotype remain unclear.

VSMCs undergo the metabolic transition from mitochondrial oxidative phosphorylation (OXPHOS) to aerobic glycolysis, which is also called as Warburg effect (6). Our previous proteomic analyses have revealed that key enzymes of aerobic glycolysis, such as pyruvate kinase isoform M2 (PKM2), phosphofructokinase-1 (PFK1), and lactate dehydrogenase-A (LDHA) were upregulated in synthetic VSMCs (7, 8, 9). In addition, compelling evidence has also shown that these rate-limiting reaction enzymes of glycolysis were associated with the development of atherosclerosis, restenosis, and intimal hyperplasia (10, 11, 12). However, the endogenous modulator in VSMCs that fine-tunes the balance between OXPHOS and glycolysis remains poorly understood.

The glycolytic enzyme phosphofructokinase, liver type (PFKL), PFK-platelet type (PFKP), and PFK-muscle type (PFKM) are isozymes of PFK1 that catalyze the phosphorylation of fructose-6-phosphate (F6P) to fructose-1,6-bisphosphate (13). The enzymatic reaction catalyzed by PFK1 generates additional ATP to meet increasing energy requirements for VSMC proliferation (14). Furthermore, a recent study has reported that the mitochondrial outer membrane protein mitofusin-2 (MFN2) mediated PFK1 degradation, therefore suppressing VSMC proliferation/migration and alleviating neointimal hyperplasia in vein grafts (15). This finding indicates the involvement of PFK1 in VSMC phenotype switching, but further investigation is needed to unravel additional regulatory mechanisms.

Acetylation is a classic PTM by the addition of acetyl groups to lysine (K) residues. Nonhistone acetylation modifications play important roles in regulating protein stability, enzyme activity and subcellular localization (16). A proteomic analysis has discovered that enzymes that participate in intermediate metabolism were preferentially acetylated. In addition, almost every enzyme in glycolysis, gluconeogenesis, TCA (the tricarboxylic acid) cycle, the urea cycle, fatty acid metabolism, and glycogen metabolism was acetylated (17). Unquestionably, PFK1 is susceptible to acetylation. Until now, few reports have revealed the effect of acetylation of PFK1 on its function and role in vascular remodeling. Thus, we wonder if acetylation of PFK1 could regulate its enzymatic activity, thereby influencing glycolysis rate and VSMC growth during VSMC phenotypic switching.

Histone deacetylases (HDACs), a family of enzymes that remove acetyl groups from lysine residues of histone proteins and nonhistone proteins, are responsible for mediating reversible lysine acetylation (18, 19). Until now, five members of HDACs family (HDAC 1, 2, 3, 4 and 6) (18, 20, 21) have been reported to control VSMC proliferation and migration. These findings raise an interesting question of whether the HDAC family is involved in the regulation of acetylation of PFK1.

Here, we report that HDAC6 is a deacetylase for PFKL. After PDGF-BB stimulation, PFKL deacetylated at K563 residue, which is located in the interface of the PFK1 dimer. TSA or short-interfering RNA-mediated knockdown of HDAC6 instead of HDAC8 significantly rescued the reduction in PFKL acetylation and subsequently inhibited VSMC proliferation. PDGF-BB-induced VSMC proliferation is significantly increased in VSMCs overexpressing the PFKL K563 R mutant relative to PFKL WT control cells, whereas that of the PFKL K563Q mutant significantly decreased based on the analysis of cell viability, cell number and the expression levels of PCNA. K563 acetylation of PFKL hinders the formation of PFKL tetramers, which results in attenuated enzymatic activity of the PFKL protein, leading to a decrease in lactate levels and the VSMC viability. Overall, these findings highlight acetylation as a classic post-translational PFKL modification that regulates its activity, inducing metabolic reprogramming and VSMC proliferation.

## Results

### PFKL is essential for VSMC proliferation

**Figure 1:**
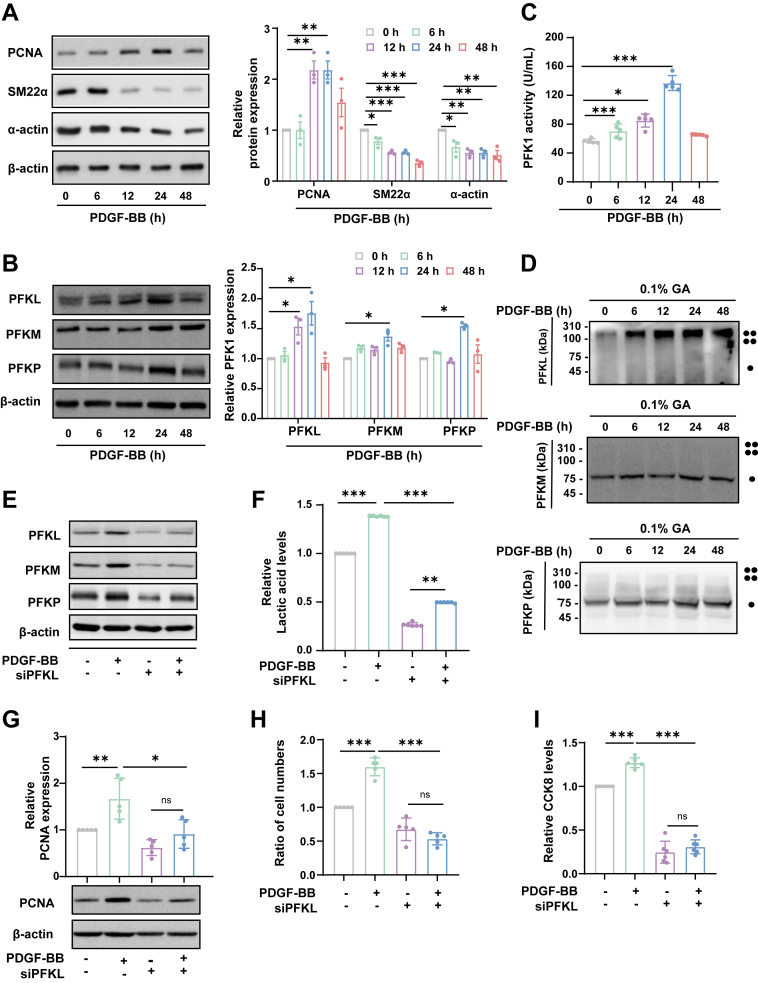
**PFKL is essential for VSMC proliferation.***A*, representative western blotting images and quantitative analysis results of PCNA, α-actin and SM22α in VSMCs treated with PDGF-BB (10 ng/ml) at different times(n = 3). *B*, representative western blotting images and quantitative analysis results of PFKL, PFKM and PFKP in VSMCs treated with PDGF-BB (10 ng/ml) at different times(n = 3). *C*, quantitative analysis of phosphofructokinase (PFK) activity in VSMCs treated with PDGF-BB (10 ng/ml) at different times(n = 5). *D*, analysis of PFKL/PFKM/PFKP tetramerization in cells using a glutaraldehyde crosslinked method(n = 3). *E*, immunoblotting analysis of PFKL/PFKM/PFKP protein expression upon depletion of PFKL using sicon/si PFKL with or without PDGF-BB stimulation for 24 h(n = 3). *F*, quantitative analysis of lactate levels in VSMCs upon depletion of PFKL using sicon/si PFKL with or without PDGF-BB stimulation for 24 h (n = 6). *G*, representative western blotting images and quantitative analysis results of PCNA protein expression upon depletion of PFKL using sicon/siPFKL with or without PDGF-BB stimulation for 24 h (n = 5). *H*, cell growth of VSMCs upon depletion of PFKL using sicon/si PFKL with or without PDGF-BB stimulation for 24 h (n = 5). *I*, cell viability of VSMCs upon depletion of PFKL using sicon/si PFKL with or without PDGF-BB stimulation for 24 h (n = 6). Data are presented as mean ± SD. One-way ANOVA was used followed by Tukey 's multiple comparisons test for statistical analysis. ∗*p* < 0.05, ∗∗*p* < 0.01, ∗∗∗*p* < 0.001, ns represents no significance.

PDGF-BB is a canonical stimulus that triggers VSMC phenotypic switching from a contractile state to a proliferative/synthetic state (22). Our previous work has shown that PDGF-BB increased PFK1 expression in proliferative/synthetic VSMCs (8). Mammalian PFK1 is composed of three different isoforms: muscle (PFKM), liver (PFKL), and platelet (PFKP). Especially PFKL has significant influence on glycolysis (23). Our proteomic analysis showed that PFKL is significantly increased in the LDLR^+/−^ hamsters restenosis model (8). This implies that PFKL may play a crucial role in the process of cascular hyperplasia. To explore whether PFK1 regulates VSMC phenotypic switching, cultured primary rat VSMCs were stimulated with PDGF-BB (10 ng/ml) for different durations (0–48 h). The levels of SM22α and α-actin, VSMC contractile marker, decreased over time upon PDGF-BB stimulation. The levels of PCNA, a marker of proliferation, gradually increased at 12 h and peaked at 24 h (Fig. 1*A*), indicating a gradual switch in VSMCs from a contractile phenotype to a synthetic/proliferating phenotype. Our results indicated PFKL/PFKM/PFKP expression was significantly increased in VSMCs at 24 h on PDGF-BB stimulation (Fig. 1*B*), accompanied by associated degrees of increased PFKL activity (Fig. 1*C*).

Generally, PFKM, PFKL, and PFKP are randomly combined into homologous or heterologous tetramers to perform their physiological functions (24). Given that the tetrameric form of PFK1 is the active enzyme form, glutaraldehyde cross-linking experiments were performed to detect the tetrameric form of PFK1. We observed a significant increase in PFKL tetramerization following stimulation with PDGF-BB for different durations, while no obvious blots were observed in the tetrameric PFKM or PFKP (Fig. 1*D*). These data suggest that both the expression and activity of PFKL are upregulated in proliferative VSMCs.

Next, we asked whether the interference of the PFKL expression affects VSMC proliferation. We found that siPFKL also reduced the expression of PFKM and PFKP (Fig. 1*E*), likely due to sequence similarity between PFKL and other isoforms. A previous study suggested that lactate may promote a synthetic phenotype in SMC (25). PFKL is known to facilitate lactate production by regulating aerobic glycolysis (26), so we wonder whether PFKL deletion in SMCs reduces lactate production. Our results indicated PFKL deficiency led to a decrease in PDGF-BB-mediated lactate production (Fig. 1*F*). Furthermore, VSMCs lacking PFKL proliferated less when compared to VSMCs containing PFKL subjected to PDGF-BB, which was confirmed by PCNA expression (Fig. 1*G*), cell numbers (Fig. 1*H*), and cell viability (Fig. 1*I*). These results suggest that the knockdown of PFKL significantly inhibits glycolysis and VSMC proliferation.

### Inhibition of PFKL acetylation promotes its activation, lactate production and VSMC proliferation

**Figure 2:**
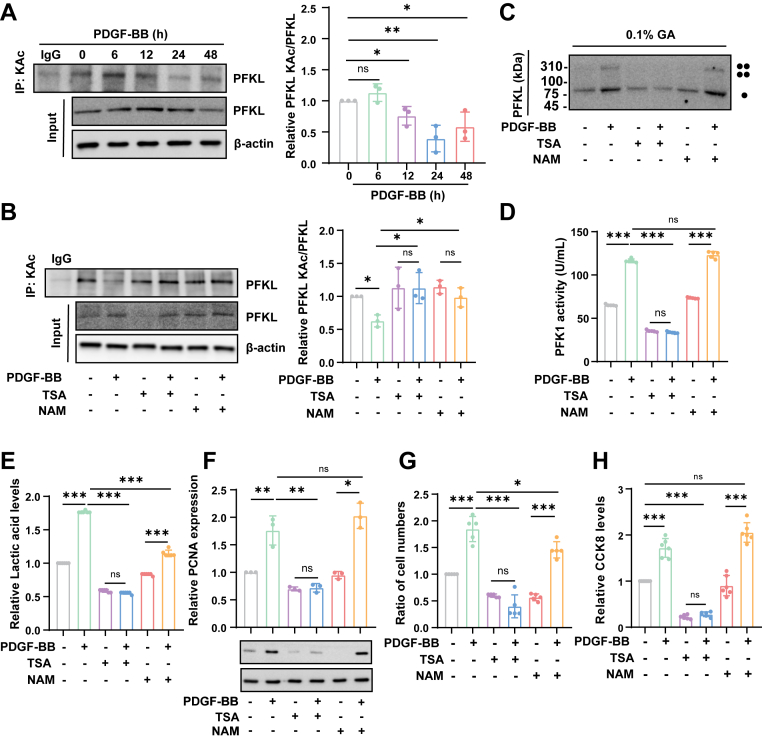
**Inhibition of PFKL acetylation promotes its activation, lactate production and VSMC proliferation.***A*, complexes immunoprecipitated with anti-acetyl-lysine antibodies were immunoblotted with anti-PFKL antibody in VSMCs with PDGF-BB treatment for different durations (n = 3). *B*, the acetylation of PFKL was shown by Western blot using IP after treatment with TSA or NAM and PDGF-BB for 24 h(n = 3). *C*, analysis of PFKL tetramerization in VSMCs treated by TSA or NAM with or without PDGF-BB for 24 h (n = 3). *D*, quantitative analysis of PFK activity in VSMCs treated by TSA or NAM with or without PDGF-BB for 24 h (n = 5). *E*, quantitative analysis of lactate levels in VSMCs treated by TSA or NAM with or without PDGF-BB for 24 h (n = 5). *F*, representative western blotting images and quantitative analysis results of PCNA protein expression after VSMCs were treated by TSA or NAM with or without PDGF-BB for 24 h (n = 5). *G*, cell mumbers of VSMCs treated by TSA or NAM with or without PDGF-BB for 24 h (n = 5). *H*, cell viability of VSMCs treated by TSA or NAM with or without PDGF-BB for 24 h (n = 6). Data are presented as mean ± SD. One-way ANOVA was used followed by Tukey's multiple comparisons test for statistical analysis. ∗*p* < 0.05, ∗∗*p* < 0.01, ∗∗∗*p* < 0.001, ns represents no significance.

Acetylation plays an important role in metabolic regulation. A liquid chromatography tandem mass spectrometry (LC/LC-MS/MS) found that almost all enzymes involved in glycolysis, gluconeogenesis, TCA cycle, urea cycle, fatty acid metabolism, and glycogen metabolism were acetylated in human liver tissue, including PFKL. In our study, the expression and activity of PFKL in proliferating VSMCs significantly increased. However, the relationship between PFKL acetylation and its activity remains unclear, so we wonder if the activity of PFKL is regulated by its acetylation. First, we examined whether PFKL acetylation levels were altered in VSMCs after PDGF-BB stimulation. Our results showed that the acetylation levels of PFKL significantly decreased at 24 h in response to PDGF-BB treatment (Fig. 2*A*). Both HDAC inhibitors TSA (Trichostatin A) and NAM (Sirtuin family inhibitor) reversed the PDGF-BB-induced reduction in PFKL acetylation (Fig. 2*B*). Only TSA alleviated the PDGF-BB-induced increase of the PFKL tetramer (Fig. 2*C*) and activity (Fig. 2*D*), while NAM had no significant effect. These data demonstrate that PFKL acetylation inhibits its enzyme activity by preventing tetramer formation. We further confirmed the effect of deacetylase inhibitors on glycolysis and VSMC proliferation. Lactate production (Fig. 2*E*), the expression of PCNA (Fig. 2*F*), cell numbers (Fig. 2*G*) and cell viability (Fig. 2*H*) were simultaneously reduced by TSA pretreatment in PDGF-BB-incubated VSMCs, while NAM did not reverse these effects. Taken together, these results indicate that PFKL acetylation can inhibit its enzymatic activity, thereby significantly inhibiting glycolysis and VSMC proliferation.

### HDAC6 mediates PFKL deacetylation

**Figure 3:**
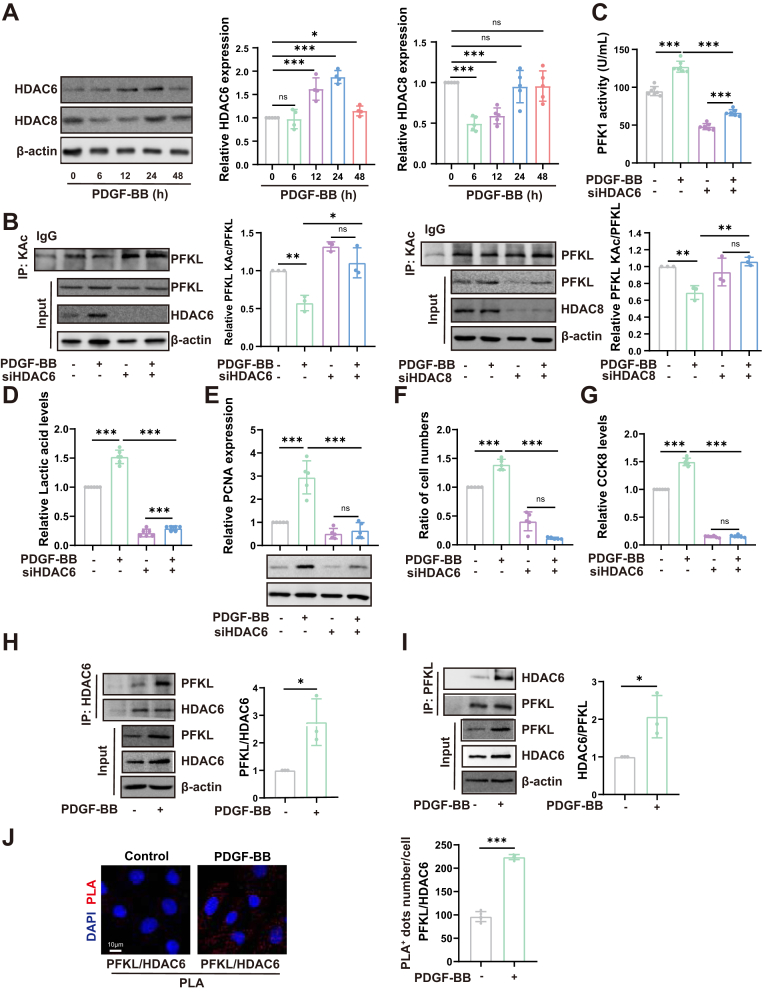
**HDAC6 mediates PFKL deacetylation.***A*, representative western blotting images and quantitative analysis results of HDAC6 and HDAC8 in VSMCs treated with PDGF-BB (10 ng/ml) at different times(n = 5). *B*, the acetylation of PFKL was examined by IP after si-HDAC6/si-HDAC8 treatment. (n = 6). *C*, quantitative analysis of PFK activity in VSMCs after si-HDAC6 treatment (n = 3). *D*, quantitative analysis of lactate levels in VSMCs after si-HDAC6 treatment (n = 6). *E*, representative western blotting images and quantitative analysis results of PCNA protein expression after si-HDAC6 treatment (n = 3). *F*, cell growth of VSMCs after si-HDAC6 treatment (n = 5). *G*, cell viability of VSMCs after si-HDAC6 treatment. *H–I*, Endogenous HDAC6-PFKL interactions in VSMCs detected by IP experiments with or without PDGF-BB stimulation for 24 h (n = 3). *J*, representative fluorescence images of PLA signals after labeling with antibodies against PFKL and HDAC6 in VSMCs with or without PDGF-BB stimulation. Positive PLA signal (*red)*, DAPI (*blue*). Bar scale = 10 μm (n = 5). Data are presented as mean ± SD. One-way ANOVA was used followed by Tukey 's multiple comparisons test for statistical analysis in (*A–G)* dara. Unpaired Student *t* test was used for statistical analysis in (*H–J*) data. ∗*p* < 0.05, ∗∗*p* < 0.01, ∗∗∗*p* < 0.001, ns represents no significance.

Studies have indicated a therapeutic role of lysine acetyltransferases and deacetylases in CVD (27). Given that TSA blocks a HDAC increasing the levels of acetylated PFKL, we hypothesized that HDACs may regulate PFKL acetylation. Studies have shown that cytosolic HDAC6 regulates endothelial cell migration and angiogenesis by deacetylating cortactin (28). HDAC8 promotes pathological cardiac growth and impairs heart function (29). In addition, in deoxycorticosterone acetate (DOCA)-salt induced chronic hypertension rats, the activity and expression of HDAC6 and HDAC8 were significantly increased (30). However, the role of HDAC6 and HDAC8 in VSMCs remains unclear. We first assessed the protein expression levels of HDAC6 and HDAC8 in proliferating VSMCs. HDAC6 expression gradually increased at 12 h with PDGF-BB stimulation, reaching a peak at 24 h, whereas HDAC8 expression decreased at 6 h and 12 h with PDGF-BB stimulation, and no change was detected on HDAC8 expression after PDGF-BB stimulation for 24 h (Fig. 3*A*). To explore the effect of HDAC6 and HDAC8 on PFKL acetylation, siRNA knockdown of HDAC6 or HDAC8 was performed (Fig. 3*B*). Only the abrogation of HDAC6 simultaneously reversed the reduction in PFKL acetylation, the increase in PFK1 activity (Fig. 3*C*), lactate production (Fig. 3*D*), PCNA expression (Fig. 3*E*), cell numbers, and cell viability (Fig. 3, *F* and *G*) upon PDGF-BB treatment (Fig. 3, *B*–*D*), demonstrating that HDAC6 plays a crucial role in regulating glycolysis and VSMC proliferation. Moreover, immunoprecipitation and the *in situ* proximity ligation assay results further confirmed that the interaction of HDAC6 with endogenous PFKL was significantly enhanced upon PDGF-BB stimulation (Fig. 3, *H*–*J*). In addition, the binding of HDAC8 to PFKL did not change in VSMCs after PDGF-BB treatment (Fig. S1). Taken together, these data suggest that HDAC6, instead of HDAC8, mediates PFKL deacetylation via direct interaction, thereby enhancing PFKL activity and promoting glycolysis in VSMCs.

### PFKL acetylation at the K563 regulates VSMC proliferation and neointimal formation

**Figure 4:**
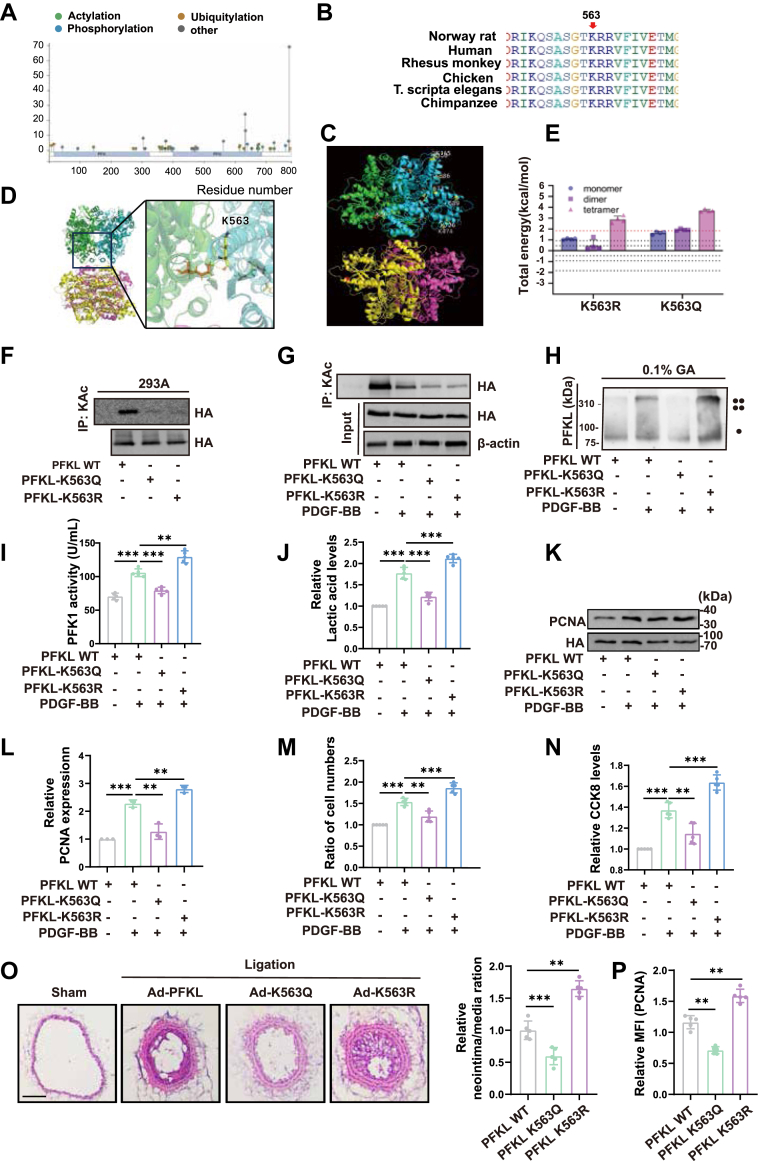
**HDAC6 regulates PFKL acetylation at the K563 to promote VSMC proliferation.***A*, Potential lysine residues susceptible to acetylation on PFKL predicted by PhosphoSitePlus v6.7.1.1 database. *B*, the sequences around potential lysine residues susceptible to acetylation of PFKL from different species were aligned by BioEdit software. Conserved lysine residues are marked in purple. *C* and *D,* the docking analysis of K563 residue on PFKL using ZDOCK software and visualized by PyMol software. *E*, the free energy of the spatial structure after K563 mutation or simulated acetylation mutation calculated by the YASARA software. *F*, analysis of PFKL acetylation in 293A cells transduced with the PFKL WT, PFKL K563Q or PFKL K563R plasmids (n = 3). *G*, analysis of PFKL acetylation in VSMCs transduced with the PFKL WT, PFKL K563Q or PFKL K563R adenovirus followed by PDGF-BB stimulation (n = 3). *H*, analysis of PFKL tetramerization in VSMCs transduced with the PFKL WT, PFKL K563Q or PFKL K563R adenovirus followed by PDGF-BB treatment. *I*, quantitative analysis of PFK activity in VSMCs transduced with the PFKL WT, PFKL K563Q or PFKL K563R adenovirus followed by PDGF-BB treatment for 24 h (n = 3). *J*, quantitative analysis of lactate levels in VSMCs transduced with the PFKL WT, PFKL K563Q or PFKL K563R adenovirus followed by PDGF-BB treatment for 24 h(n = 4). *K–L*, representative western blotting images and quantitative analysis results of PCNA protein expression in VSMCs transduced with the PFKL WT, PFKL K563Q or PFKL K563R adenovirus followed by PDGF-BB treatment for 24 h(n = 3). *M*, cell growth of VSMCs transduced with the PFKL WT, PFKL K563Q or PFKL K563R adenovirus followed by PDGF-BB treatment for 24 h (n = 5). *N*, cell viability of VSMCs transduced with the PFKL WT, PFKL K563Q, or PFKL K563R adenovirus followed by PDGF-BB treatment for 24 h (n = 5). *O*, hematoxylin and eosin staining analysis of ligation-injured carotid arteries transduced with the PFKL WT, K563Q, or K563R adenovirus at day 14. Bar scale = 50 μm (n = 5). *P*, immunofluorescence staining analysis of PCNA in injured carotid arteries transduced with the PFKL WT, K563Q, or K563R adenovirus (n = 5). Data are presented as mean ± SD. One-way ANOVA was used followed by Tukey 's multiple comparisons test for statistical analysis. ∗*p* < 0.05, ∗∗*p* < 0.01, ∗∗∗*p* < 0.001, ns represents no significance.

To decipher potential lysine residues susceptible to acetylation in PFKL, we used the PhosphoSitePlus v6.7.1.1 (https://www.phosphosite.org/) database to predict potential acetylation sites on PFKL. Ten lysine residues were predicted to undergo acetylation, with K360, K365, K383, K386, K474, K563, K677, K689, K726, and K727 as the candidate sites (Fig. 4*A*). PTMs in highly conserved regions of protein sequence evolution may indicate key regulatory roles in biological processes. Therefore, BioEdit sequence alignment software was applied to analyze the conserved properties of these 10 sites and their adjacent amino acid sequences. Sequence alignment analysis revealed that the surrounding amino acid sequences of the remaining seven sites were highly conserved except for K383, K386, and K474 (Fig. 4*B*). Generally, eukaryotic PFKL tetramerizes *via* its regulatory domains, and oligomerization regulates PFKL activity. However, whether PFKL acetylation occurs in its regulatory domain or between the monomers of the PFKL dimer is unknown. Therefore, we analyzed the positions of the remaining seven conserved residues in the PFK1 higher-order structure and found that the K563 residue is located exactly at the interface of the PFKL dimer (Fig. 4, *C* and *D*). Next, the YASARA software was used to calculate the free energy of PFKL spatial structure after the K563 deletion or simulated acetylation mutation. We found that the free energy of the spatial structure changed significantly after the K563 mutation (Fig. 4*E*), indicating that the acetylation of PFKL K563 may be related to the formation of the PFK1 dimer and the stability of the tetramer. To confirm our hypothesis, we replaced K563 with either nonacetylated-mimeticarginine (K563R) or acetyl-mimetic glutamine (K563Q) to construct PFKL mutants.

HA-PFKL WT, PFKL K563Q (simulated acetylation), and K563R (simulated deacetylation) plasmids were transferred into 293A cells for 48 h, IP experiments were performed and results showed that K563 mutation significantly reduced the PFKL acetylation (Fig. 4*F*). To further verify the functional impact of K563 acetylation of PFKL *in vitro*, recombinant adenoviral vectors were used to transduce VSMCs with HA-PFKL WT, PFKL K563Q or K563R followed by PDGF-BB stimulation. When Ad-PFKL WT, Ad-PFKL K563Q and Ad-PFKL K563R were re-expressed in VSMCs in which endogenous PFKL expression was knocked down by anti-PFKL siRNA, the acetylation level of PFKL markedly reduced in the K563R and K563Q mutants compared with that of the wild-type PFKL (Fig. 4*G*), indicating PFKL-K563 was the major acetylation site upon PDGF-BB challenge. Western blotting confirmed that acetylation of PFKL was undetectable in the PFKL K563Q or K563 R group in the absence of PDGF-BB (Fig. S2*A*). However, no significant phenotypic changes were observed in VSMCs 48 h after adenovirus-mediated gene transfer in the absence of PDGF-BB (Fig. S2, *B* and *C*). These results demonstrated that K-to-R or Q mutation of PFKL does not affect physiological function of VSMCs. Glutaraldehyde-crosslinking experiments showed that PFKL K563Q mutation to mimic PFKL acetylation evidently decreased PFKL oligomerization and its activity. On the contrary, PFKL K563R mutation to mimic PFKL deacetylation significantly increased the formation of PFKL tetramers and activity (Fig. 4, *H* and *I*). Again, the recombinant adenoviral vector carrying the PFKL K563R mutant aggravated, while the K563Q mutant attenuated lactate production (Fig. 4*J*), the expression of PCNA (Fig. 4, *K* and *L*), cell number (Fig. 4*M*) and cell vitality (Fig. 4*N*) in VSMCs in response to PDGF-BB. To further probe whether PFKL deacetylation at K563 contributes to intimal hyperplasia *in vivo*, we performed periadventitial infection of adenoviruses encoding PFKL WT, K563Q, or K563R in carotid arteries. Fourteen days after ligation, adenovirus-mediated PFKL WT, K563Q, or K563R was verified by immunofluorescence results of HA (Fig. S4*A*). The intimal hyperplasia degree obviously decreased in PFKL K563Q expressing arteries, whereas it significantly increased in PFKL K563R expressing arteries compared with the PFKL WT expressing arteries (Fig. 4*O*). In parallel, a reduction in PCNA was also observed in arteries overexpressing PFKL K563Q mutant, whereas significantly increased in PFKL K563R expressing arteries relative to PFKL WT control arteries (Figs. 4*P* and S4*B*). Taken together, these results suggest that PFKL deacetylation at K563 increases tetrameric form of PFKL, thereby increasing it's enzymatic activity and activating glycolysis, ultimately facilitating the proliferation of VSMCs and neointimal formation.

## Discussion

Upon vascular injury, such as angioplasty or bypass surgery, VSMCs dedifferentiate from a “contractile” phenotype to a highly “synthetic” phenotype characterized by an enhanced rate of proliferation, migration, production of extracellular matrix (ECM) components, and reduction of VSMC-specific markers, including smooth muscle myosin heavy chain (SM-MHC), smooth muscle 22α (SM22α), and calponin (31, 32). Synthetic VSMCs demonstrated an increased glycolytic flux, with a decrease in glucose oxidation (33). This metabolic reprogramming provides VSMCs a growth advantage by fueling their proliferation. In parallel, our recent research also validated that the levels of several rate-limiting glycolytic enzymes, such as PFKL(8), PKM2(7), and LDHA(9), were significantly upregulated in PDGF-BB stimulated VSMCs as compared to those in the control group. PFK1, as an enzyme involved in the first rate-limiting step of glycolysis, typically functions as a tetramer. PFKP, PFKL and PFKM are three different PFK1 isoforms in humans (24). PFKP expression is significantly elevated in pulmonary arterial hypertension patient SMCs (11) and hypertrophic cardiomyocytes (34). Additionally, PFKM plays a critical role in regulating cardiomyocyte proliferation and cardiac toxicity (35). PFKL can be activated by TAp73, thereby promoting glycolysis and enhancing cell proliferation in tumor cells (36). These findings highlight the important role of PFK-1 in cell proliferation.

The activity of PFKL is regulated by PTMs, such as ubiquitination (15), phosphorylation (37) and glycosylation (38). These PTMs have significant effects on PFKL activity under both physiological and pathophysiological conditions, making them potential targets for metabolism-related diseases. Recent studies have implicated a fundamental role of reversible protein acetylation in the regulation of CVDs such as hypertension (39, 40), diabetic cardiomyopathy (41), coronary artery disease (42), pulmonary arterial hypertension (PAH) (43) and heart failure (44). The acetylation modification of PFKL was reported as early as 2010 (16). Acetylation of PFKL is a prerequisite for the formation of the multienzyme metabolic complex (45). However, how PFKL acetyl modifications affect cardiovascular pathophysiology has not been extensively explored.

In this work, elevated levels of PFKL were detected in synthetic VSMCs stimulated with PDGF-BB, whereas the acetylation of PFKL was decreased in PDGF-BB induced proliferative VSMCs. This piqued our interest in focusing on the relationship between PFKL acetylation and its activity. Despite predictive analysis indicating that PFKL has 10 potential acetylation modification sites, three-dimensional structural screening predicted that lysines 563 and 689 may influence PFKL activity. The 3-dimensional structure of PFKL indicates that K563 is located in the interface of the PFKL dimer. Our IP results identified K563 as an important regulatory acetylation site in PFKL. Moreover, the PDGF-BB-induced tetramer formation of PFKL, lactate formation and VSMC growth were decreased in PFKL K563Q expressing cells, whereas these were increased in PFKL K563R expressing cells. Thus, these findings suggest that acetyl modification at the K563 site enables PFKL to acquire the ability to facilitate PFKL activation.

HDAC6, a histone deacetyltransferase, plays a pivotal role in regulating microtubule dynamics, DNA damage response and apoptosis (46). Previous studies have highlighted the effect of HDAC6 on VSMC proliferation and migration (20). We discovered that PFKL can be deacetylated at lysine 563 by HDAC6 to increase its enzymatic activity, thereby promoting lactate production and proliferation of VSMCs. Our study identified the HDAC6/PFKL axis as a pivotal regulatory pathway for the Warburg effect in VSMCs. This study offers a molecular explanation linking the regulatory role of HDAC6 in driving the growth and progression of VSMCs to its function in promoting glycolysis. Moreover, our findings establish a connection between HDAC6, the Warburg effect, and VSMC growth, demonstrating that HDAC6 promotes VSMC growth and metastasis by inducing PFKL-mediated Warburg effect.

## Experimental procedures

### Cell cultures

The thoracic aorta was collected from male Sprague-Dawley rats weighing 60 to 80 g and then cut into pieces to adhere them in the flasks after removal of the adventitia.

VSMCs were cultured in Dulbecco's-modified Eagle's medium (DMEM) (Invitrogen) with 1 g/L glucose containing 10% fetal bovine serum (FBS), 100 U/ml penicillin and 100 μg/ml streptomycin, and kept at 37 °C with 5% CO_2_. Cells were passaged after reaching confluence with 0.25% trypsin-3 mM EDTA (Sigma). Primary VSMCs from the second to the fifth passages were used in this study. The cells at 70% to 80% confluence were treated with PDGF-BB (10 ng/ml) (R&D Systems Inc.) for further culture. This study was performed *via* a protocol approved by the Institutional Animal Care and Use Committee of Hebei Medical University, in accordance with the Guide for the Care and Use of Laboratory Animals, and the Hebei Medical University Clinical Research Ethics Committee.

### siRNA transfection

Small interfering siRNAs that targeting rat PFKL/HDAC6/HDAC8 and scrambled siRNA served as control siRNA were designed and synthesized by GenePharma. Transfection of rat VSMCs with siRNAs *in vitro* was performed by using Lipofectamine RNAiMIX reagent (GenePharma) according to the manufacturer's protocol. 6 h after transfection, VSMCs were incubated in a medium containing 3% FBS for 18 h, then stimulated by PDGF-BB for 24 h. The siRNA sequences used were as follows:

siCon-S (5′-3′): UUCUCCGAACGUGUCACGUTT

siCon-AS (5′-3′): ACGUGACACGUUCGGAGAATT

si-PFKL-Rat-S (5′-3′): GCUCAGAACUACGCACACUTT

si-PFKL-Rat-AS (5′-3′): AGUGUGCGUAGUUCUGAGCTT

si-HDAC6-Rat-S (5′-3′): GCACCUAUGAUUCCGUUUATT

si-HDAC6-Rat-AS (5′-3′): UAAACGGAAUCAUAGGUGCTT

si-HDAC8-Rat-S (5′-3′): GGCAUAAACAAAUGAGGAUATT

si-HDAC8-Rat-AS (5′-3′): UAUCCUCAUUUGUUUAUGCTT.

### Cell adenovirus infection

The VSMCs were infected with the Ad-PFKL-WT, Ad-PFKL-K563R, or Ad-PFKL-K563Q adenovirus in DMEM containing 3% FBS. After 10 h, the medium was replaced with fresh medium DMEM containing 3% FBS for another 14 h, then stimulated by PDGF-BB in medium without FBS and antibiotics, and further cultured for 24 h.

### Western blotting analysis

VSMCs were collected and lysed with NP-40 lysis buffer (50 mmol/L Tris– HCl, pH 7.5, 150 mmol/L NaCl, 1% NP-40, 1 mmol/L EDTA, 0.11% sodium butyrate) for 30 min on ice. The lysate was centrifuged at 12,000 rpm for 10 min at 4 °C, and the supernatant was collected for subsequent experiments. Equal amounts of protein (20–100 μg) were resolved by 10% SDS-PAGE gel and then transferred to PVDF membranes (Millipore). After blocking with 5% nonfat milk in Tris-buffered saline containing 0.1% Tween-20 (TBST) for 1 h at room temperature, membranes were incubated with specific primary antibody overnight at 4 °C. Antibodies used in this study were obtained from the following sources: anti-PFKL antibody (Abcam, ab97443), anti-PFKM antibody (Abcam, ab154804), anti-PFKP antibody (Abcam, ab204131), anti-HDAC6 antibody (Abcam, ab239362), anti-HDAC8 antibody (Abcam, ab274372) anti-PCNA antibody (Proteintech, 10205-2-AP), anti-Kac antibody (Arigobio, ARG57185), anti-HA antibody (MBL, M180–3), anti-SM22α antibody (Abcam, ab14106), Anti-α-actin (PTM Biolabs, PTM-5216), and anti-β-actin antibody (Proteintech, 66009-1-Ig). After washing, the membranes were incubated with horseradish peroxidase (HRP)-conjugated secondary antibodies (1:10,000; Proteintech) for 1 h at room temperature. Immunoblots were visualized with the ECL detection system and quantified with Image Studio software.

### Immunoprecipitation assay

Cells were lysed as described. 20 μg of each supernatant was used as input. Approximately 40 μg of the clarified cell lysate were incubated with 5 μl indicated antibodies for at least 2 h with rocking at 4 °C, followed by incubation with 20 μl protein A/G agarose (Santa Cruz Biotechnology, sc-2003) overnight at 4 °C. The samples were then centrifuged at 2000 rpm/min at 4 °C for 5 min, the supernatant was discarded, and the pellet was washed by NP-40 buffer for three times. Finally, the bound protein and the input were analyzed by Western blot analysis.

### Evaluation of VSMC proliferation

VSMCs' proliferation was evaluated with PCNA protein expression, CCK8 cell viability assay and Cell counting. The PCNA protein expression was examined with a Western blot assay. For the CCK8 cell viability assay, cells were seeded into 96-well plates, and CCK-8 test fluid (Report, RP-RC3028) was added after the indicated treatment. After incubating for 1 h at 37 °C, the absorbance was recorded at a wavelength of 450 nm in a microplate reader. The cell counting was performed using our previous methods (9).

### Glutaraldehyde crosslink assay

700 μg protein solution was added to a tube and replenished to 500 μl with NP40 buffer, 50% glutaraldehyde stock solution (Report, RS0024) was added to the mixture, the final concentration of glutaraldehyde was 0.1%, and the mixture was fixed on ice for 10 min. Then the fixation was terminated with 5× Launmi buffer (0.25 M pH 6.8 Tris-HCl, 10% SDS, 0.5% bromophenol blue, 50% glycerol, 2.71% DTT) and boiled for 5 min. Western blot analysis was performed subsequently as previously introduced.

### PFKL activity assay

PFKL activity was determined using the rate of ADP production *via* auxiliary enzymes pyruvate kinase (PK) and lactate dehydrogenase (LDH) adapted to a 96-well format. The PFKL activity was correlated with the consumption of NADH, which can be determined by the decrease in the absorbance at 340 nm. The auxiliary enzymes and reagents were provided by the PFK activity testing kit (Boxbio, AKSU062 M) according to the manufacturer's protocol. Each reaction well (96-well) contains 10 μl auxiliary enzymes, 10 μl reagent 1, 10 μl reagent 2, 170 μl reagent 3 and 200 μl samples. Absorbance at 340 nm was measured at 20 s (A1) and 600 s (A2), and the PFK1 activity was calculated using the formula provided in the kit.

### Construction and transfection of pCMV-HA-PFKL plasmids

pCMV-HA-PFKL wide type (WT) and site mutation plasmids were generated by SyngenTech (Beijing Syngentech Co., LTD). The coding sequence (CDS) of the target gene was queried and mutated to complete the plasmid construction. The plasmid was identified by enzymatic cleavage and sequencing of the target gene using restriction endonucleases to obtain the correct plasmid construction. The pCMV-HA- PFKL plasmids were transfected into HEK293A (Procell Life Science&Technology Co., Ltd) cells with transfection reagent (Invitrogen) for 48 h.

### Construction and infection of recombinant adenovirus

The pAdTrack-CMV vector was used to amplify the target gene fragment by PCR, and after double enzyme digestion, the vector and target gene were linked and identified after transformation. Finally, the adenovirus was packaged and purified in HEK293A cells (Hanbio Co.LTD). Then the recombinant adenoviral vectors encoding haemagglutinin (HA) -tagged constitutively active PFKL WT (Ad-PFKL-WT), PFKL K563Q (Ad-PFKL-K563Q) or PFKL K563R (Ad-PFKL-K563R) were transfected into VSMCs.

### Carotid artery ligation model in mice and adenovirus infection

The left carotid artery of male C57BL/6J mice was ligated with a 6-0 silk suture before the common carotid bifurcates after abdominal anesthesia. The common carotid artery was dissected free of the surrounding connective tissue and Ad-PFKL-WT (wild type), Ad-PFKL-K563Q, or Ad-PFKL-K563R (1 × 10^10^ pfu/ml) was suspended together in 20 μl pluronic F127 gel (Sigma-Aldrich; 25% wt/vol) and applied around the carotid artery for 30 min. Carotid arteries were harvested 14 days after ligation. Frozen arterial segments were sectioned at 5 μm and stained with hematoxylin and eosin and were examined by a light microscope (Nikon).

### Proximity ligation assay (PLA)

Proximity ligation assay (PLA) was conducted following the manufacturer's instructions using an assay kit (60,014, NaveniFlex). VSMCs were fixed with 4% paraformaldehyde for 15 min at room temperature. Subsequently, the cells were subjected to primary antibody incubation, followed by incubation with PLA probes (anti-mouse and anti-rabbit IgG antibodies conjugated with oligonucleotides). Ligation and amplification steps were carried out according to the manufacturer's instructions. Confocal images were acquired using a confocal microscope equipped with a 100× oil immersion objective lens.

### Statistical analysis

Data is analyzed using SPSS 21.0 and GraphPad Prism 7. Differences between the two groups were assessed using student's t-tests. Repeated measures were analyzed using analysis of variance (ANOVA). When the data did not meet assumptions of normality, multivariate analysis or degrees of freedom adjustments were performed. Data are presented as mean ± SEM, with *p* < 0.05 considered statistically significant.

## Data availability

The original dataset of this paper can be obtained from the corresponding author upon reasonable request.

## Supporting information

This article contains supporting information.

## Disclaimers

The content is solely the responsibility of the authors and does not necessarily represent the official views of the National Institutes of Health.

## Conflict of interest

The authors declare that they do not have any conflicts of interest with the content of this article.
