# Supplementary material for: HDAC6-mediated PFKL deacetylation enhances aerobic glycolysis and promotes VSMC proliferation
Source: J Biol Chem. 2025 Dec 18;302(2):111075. doi: 10.1016/j.jbc.2025.111075 (PMC12828377; doi:10.1016/j.jbc.2025.111075)
Supplement: Supplementary Materials [file mmc1.docx]

**Supplementary Materials for**

**HDAC6-mediated PFKL deacetylation enhances aerobic glycolysis and promotes VSMC proliferation**

Zhao-Kun Hu^#^, Zhi-Yan Ren^#^, Jie-Xin Pang, Hui Li, Meng-Nan Yang, Li-Hua Dong*

Correspondence to: donglihua@hebmu.edu.cn

**This file includes:**

Figures. S1 to S4

***Supplementary Figures S1-S4***


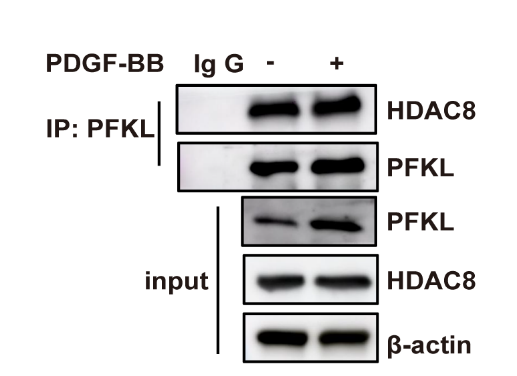


**Figure. S1.**

Endogenous PFKL-HDAC8 interactions in VSMCs with or without PDGF-BB treatment detected by coimmunoprecipitation (IP) experiments(n=3).


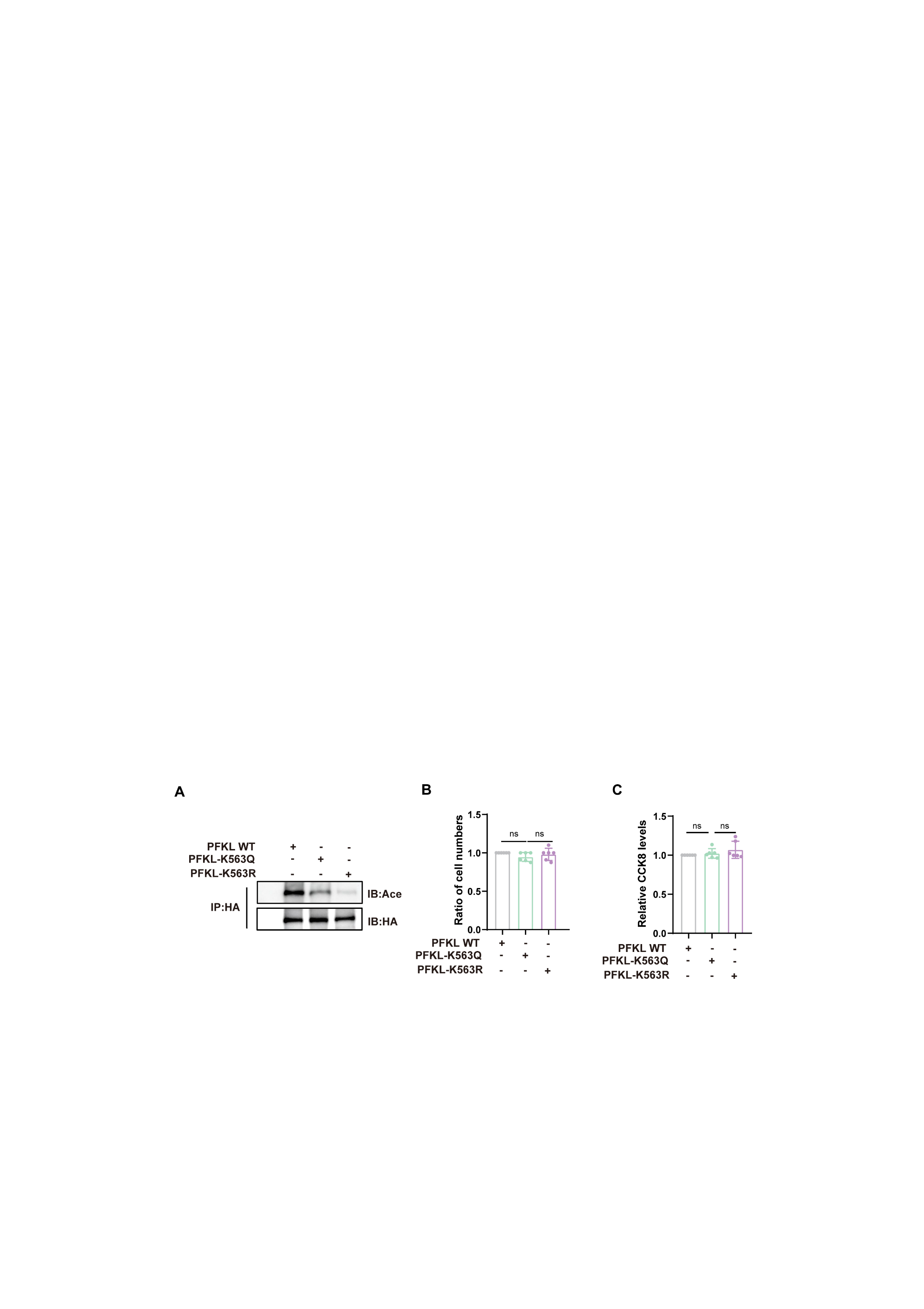


**Figure. S2.**

(A) The immunoblots show the levels of PFKL acetylation in WT, K563Q or K563R expressing VSMCs under physiological condition(n=3). (B) Cell growth of VSMCs in WT, K563Q or K563R expressing VSMCs under physiological condition(n=6). (C) Cell viability of VSMCs in WT, K563Q or K563R expressing VSMCs under physiological condition(n=6). Data are presented as mean±SD. One-way ANOVA was used Tukey’s multiple comparisions test for statistical analysis. ns represents no significance.


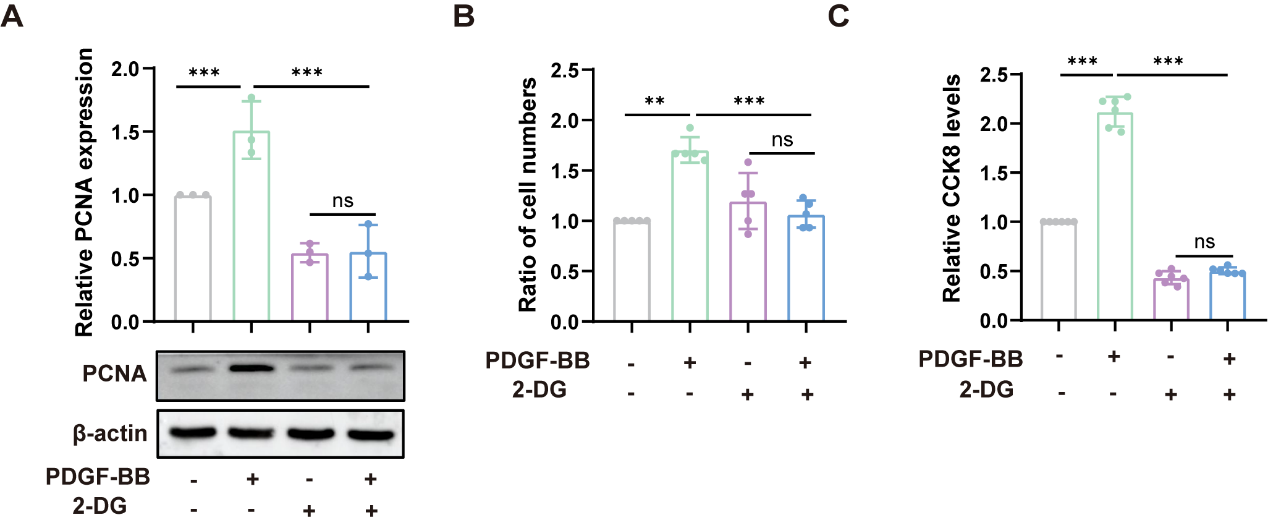


**Figure. S3.**

(A) Representative western blotting images and quantitative analysis results of PCNA protein expression after 2-DG treatment(n=3). (B) Cell growth of VSMCs after 2-DG treatment(n=5). (C) Cell viability of VSMCs after 2-DG treatment(n=6). Data are presented as mean±SD. One-way ANOVA was used Tukey’s multiple comparisions test for statistical analysis. ns represents no significance.


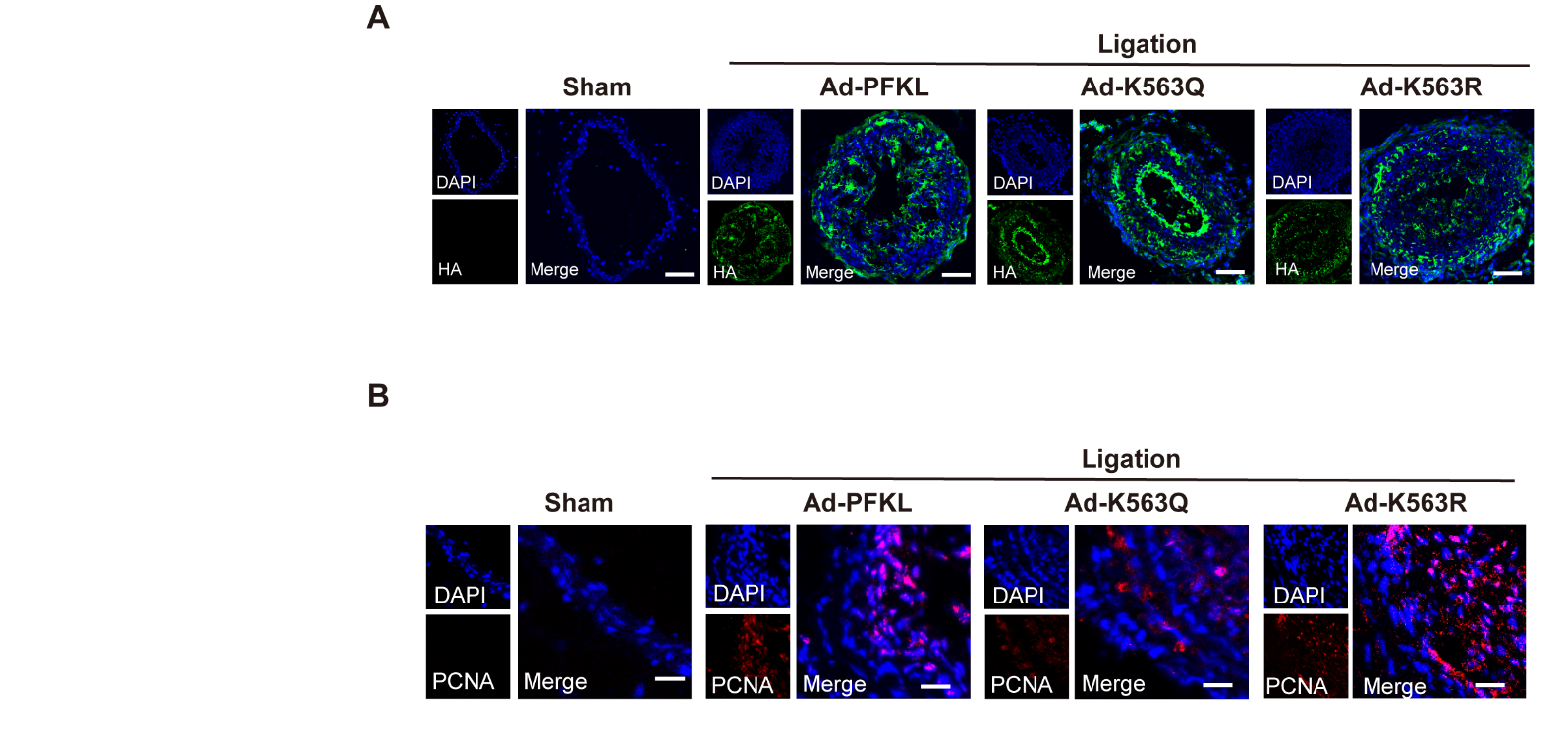


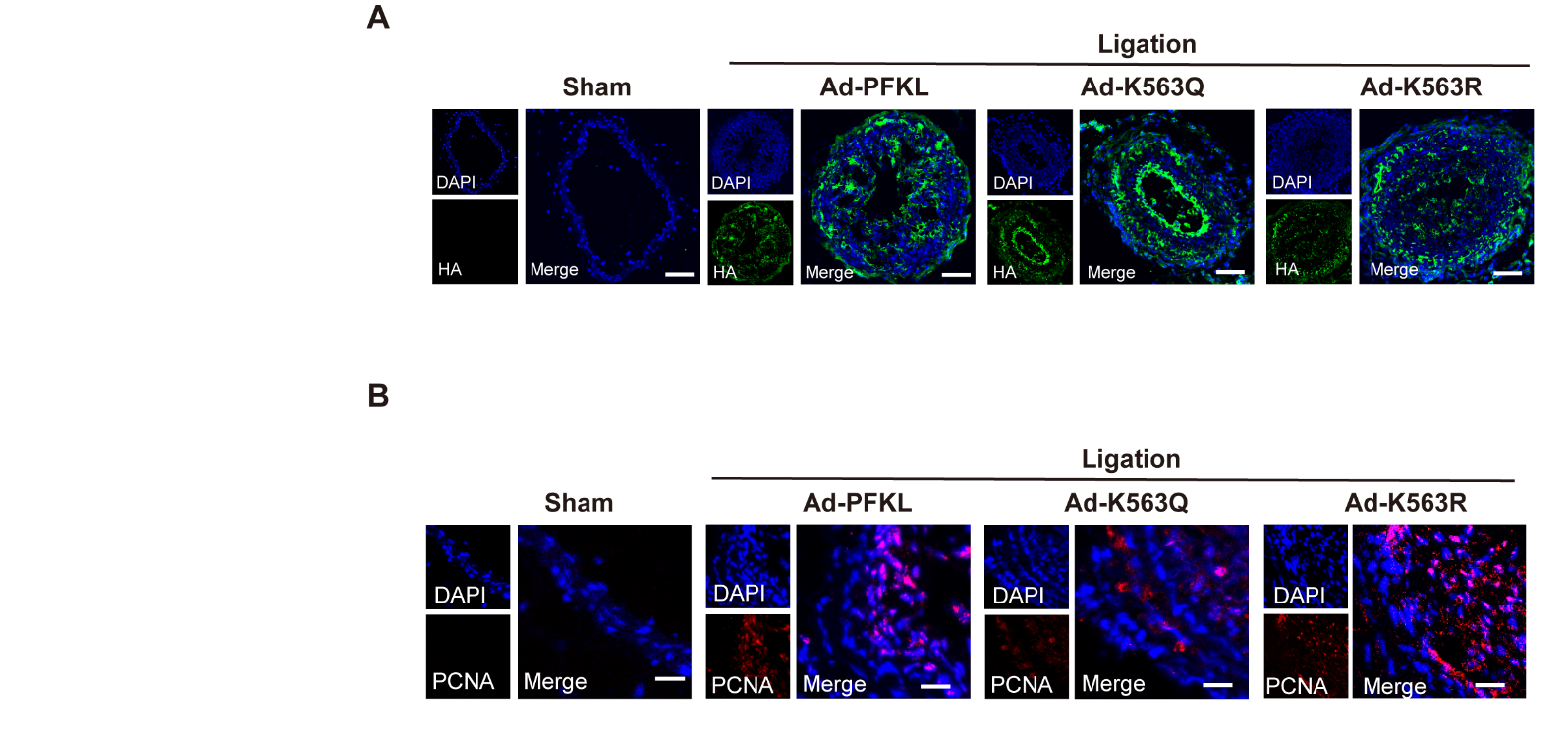


**Figure. S4.**

(A) Representative cross-section of HA following immunofluorescence (IF) staining after ligation surgery of carotid for 14 days. Scale bar=20 µm. (n=5) (B) Representative cross-sections of PCNA following IF staining after ligation surgery of carotid for 14 days. Scale bar=20 µm. (n=5)
